# Network Motifs Capable of Decoding Transcription Factor Dynamics

**DOI:** 10.1038/s41598-018-21945-2

**Published:** 2018-02-26

**Authors:** Zongmao Gao, Siheng Chen, Shanshan Qin, Chao Tang

**Affiliations:** 1 Center for Quantitative Biology, Peking University, Beijing, 100871 China; 2 Department of Physics, Tsinghua University, Beijing, 100084 China; 3 School of Physics, Peking University, Beijing, 100871 China; 4 Peking-Tsinghua Center for Life Sciences, Peking University, Beijing, 100871 China

## Abstract

Transcription factors (TFs) can encode the information of upstream signal in terms of its temporal activation dynamics. However, it remains unclear how different types of TF dynamics are decoded by downstream signalling networks. In this work, we studied all three-node transcriptional networks for their ability to distinguish two types of TF dynamics: amplitude modulation (AM), where the TF is activated with a constant amplitude, and frequency modulation (FM), where the TF activity displays an oscillatory behavior. We found two sets of network topologies: one set can differentially respond to AM TF signal but not to FM; the other set to FM signal but not to AM. Interestingly, there is little overlap between the two sets. We identified the prevalent topological features in each set and gave a mechanistic explanation as to why they can differentially respond to only one type of TF signal. We also found that some network topologies have a weak (not robust) ability to differentially respond to both AM and FM input signals by using different values of parameters for AM and FM cases. Our results provide a novel network mechanism for decoding different TF dynamics.

## Introduction

**Figure 1:**
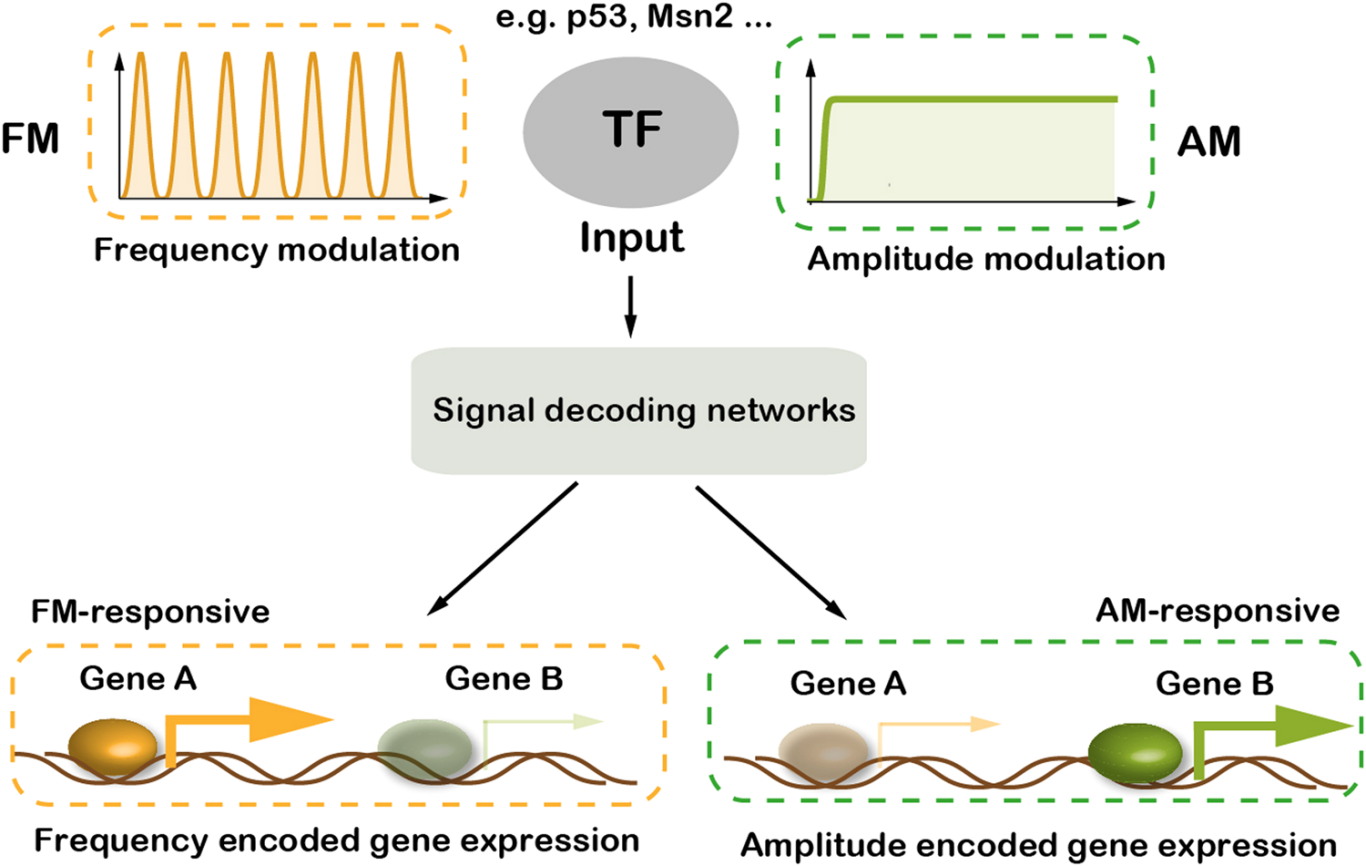
Different types of transcription factor dynamics can induce different sets of genes. Upstream stimuli can be encoded in the dynamics of transcription factors (TFs), such as amplitude modulation (AM) and frequency modulation (FM), which can be decoded and trigger the expressions of distinct target genes. Some genes are FM-responsive, with much higher expression level under FM input than under AM input, while some genes behave inversely, and are defined as AM-responsive.

In response to external and internal stimuli, such as nutritional scarcity, division or differentiation signals, DNA damage and oxidative stress, cells use signaling networks to sense, integrate and transduce upstream stimuli to transcription factors (TFs), which then trigger suitable downstream gene expression programs^1–6^. A growing number of studies have shown that information within upstream stimuli can be encoded in the types, concentrations, translocation, and dynamics of TFs^1,7^. Specially, the dynamic properties of TFs have been shown to play an important role in controlling cellular behaviors^1,6,7^. Amplitude modulation (AM) and frequency modulation (FM) (also named sustained and pulsatile signals) are two main types of TF dynamics (Fig. 1). AM is generally believed to transmit information in signaling pathways and genetic networks in a proportional way; while FM can represent a wide dynamic range of input signals and coordinates gene expressions with high accuracy^8–16^. Both types of TF dynamics are prevalent in the signaling pathways of different species^1,7,17–20^. For example, in *Saccharomyces cerevisiae*, oxidative stress leads to nuclear sustained enrichment of the TF Msn2, while glucose limitation triggers bursts of nuclear Msn2^21^. TFs in mammalian cells also exhibit dynamic behaviors, such as nuclear factor kappa B (NF-kB) in the immune response, where bacterial lipopolysaccharides lead to its sustained nuclear localization, while tumor necrosis factor-α leads to repeated pulses of nuclear localization of NF-kB^22,23^.

The dynamics of several mammalian TFs show an association with cell fate decision^1,7^. For instance, the nuclear level of the TF p53 exhibits oscillation and cell cycle arrest in response to γ-irradiation, while it remains at a sustained high level with apoptosis in response to UV radiation^19,24^. Similarly, the extracellular signal-regulated kinases (ERK) shows a pulsatile dynamics resulting in cell proliferation in response to epidermal growth factor, while ERK shows a sustained dynamics, which leads to cell differentiation in response to nerve growth factor^17^. These two types of TF dynamics trigger the expression of different genes, enabling cells to make suitable fate choices.

Although, in many cases it is clear how cells encode the information from different external stimuli into TF dynamics^1,7^, how downstream genes decode such information remains largely unknown. Recent studies have shed light on how the different promoter kinetics affects the process of decoding TF dynamics^25–28^. However, it is well known that downstream genes can be regulated by other transcription cofactors, who themselves can be regulated by upstream TFs, forming regulatory networks^29^. Whether different TF dynamics can be differentially decoded by the “read-out” networks is the focus of the paper.

To this end, we systematically explored all transcriptional regulatory networks up to three nodes. By using analytic and computational means, we identified and analyzed all network motifs that are capable of performing differential read-outs of upstream AM and FM inputs. We found that two-node networks (a transcriptional factor directly regulating its target gene) had very limited ability for the desired function. With an extra regulatory node, some three-node networks can function as efficient and robust decoders for AM or FM signal. We also identified networks that can differentially respond to both AM and FM signals (though not very robust) by using different values of parameters.

## The Model

**Figure 2:**
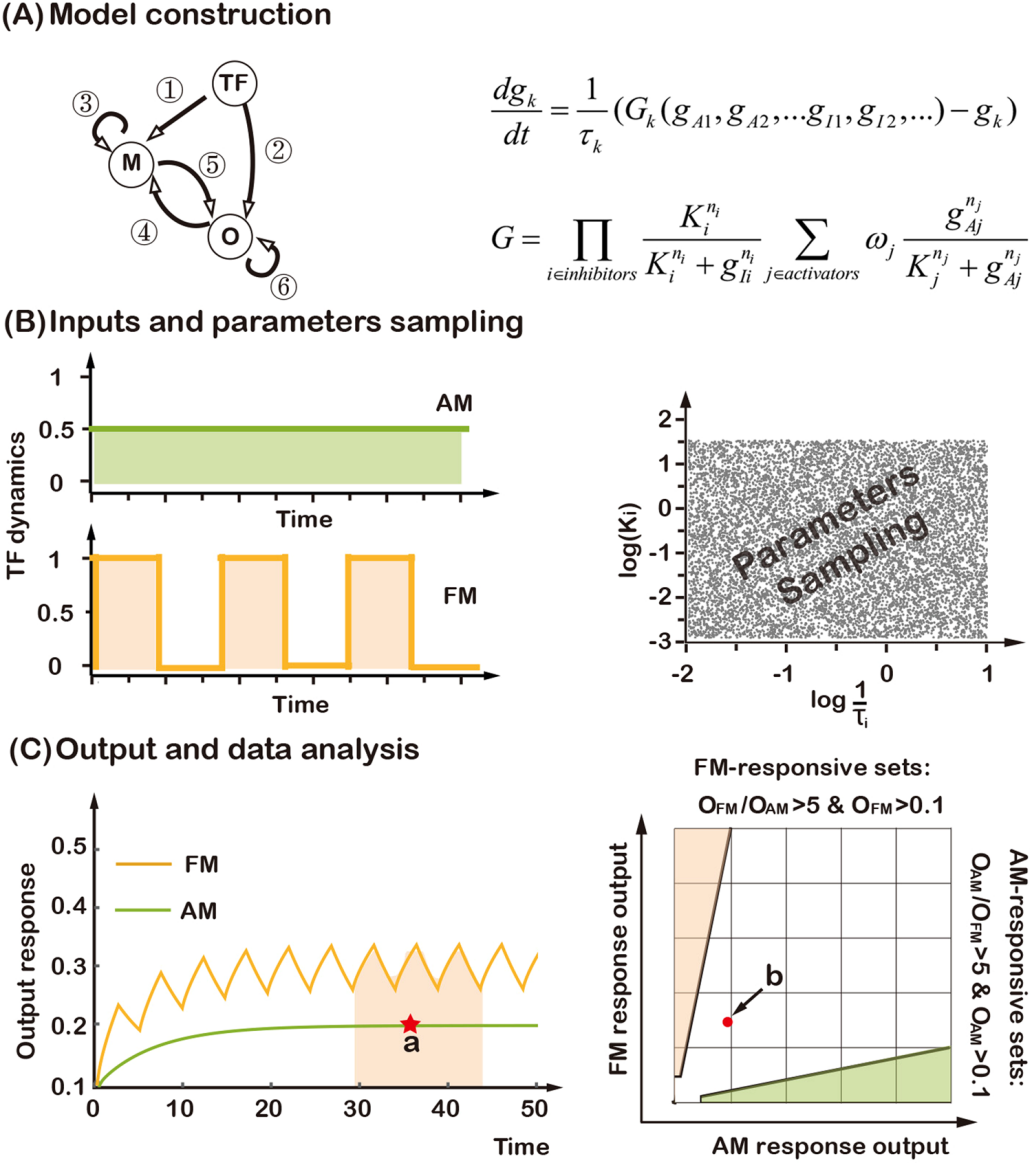
Searching for topologies that can differentially decode different transcription factor dynamics. (**A**) Possible direct links among three nodes (left) and the mathematical model (right). Each link can be in one of the three states: activation, inhibition, or no regulation. (**B**) AM and FM input dynamics of transcription factor (left) and Latin Hypercube sampling of the parameters of the model (right). (**C**) For AM, the steady-state output (a) is recorded. For FM, the asymptotic average output (schematically shown within the colored area) is recorded in the simulations (left). Illustration of the output analysis and the criterion for decoding AM- and FM-inputs (right), Red dot (b) represents the output from the example of the left panel.

In our transcriptional network topology (Fig. 2A), the node TF represents the signaling transcriptional factor with the intended dynamics. Edges between nodes represent positive or negative transcriptional regulation. All possible edges are allowed except feedback loops to TF. We modeled the effect of each edge with the Hill function. For example, for regulation by node TF, the effect of activation of its target gene is $\frac{T{F}^{n}}{T{F}^{n}+{K}^{n}}$, and that of inhibition is $\frac{{K}^{n}}{T{F}^{n}+{K}^{n}}$, where *K* represents the level of TF at half-activation or half-repression threshold, and *n* is the Hill coefficient.

Suppose *g*_*k*_ is the gene expression level of node *k*. The ordinary differential equation describing its expression can be generally written as^30^:

1 $$\frac{d{g}_{k}}{dt}=\frac{1}{{\tau }_{k}}({G}_{k}({g}_{A1},{g}_{A2},\mathrm{...}{g}_{I1},{g}_{I2},\mathrm{...})-{g}_{k})$$

where *τ*_*k*_ is the half-life of the protein, and the subscripts of *A* and *I* represent activator and inhibitor. *G* is the overall effect of different activators and inhibitors.

When multiple factors regulate one gene, they follow certain rules which determine the combined effect. The rules depend on promoter structure, interactions between TFs and the RNA polymerase. We adopted the commonly-used “strong inhibition” rule^31,32^, which assumes an additive effect of activators and a strong inhibitory effect of inhibitors (Fig. S1). Specifically, this rule sums up the activating Hill functions and multiplies them by all inhibitory Hill functions^30^. Therefore, *G* can be explicitly written as (Fig. S1A):

2 $${G}_{k}=\prod _{i\in inhibitors}\frac{{K}_{i}^{{n}_{i}}}{{K}_{i}^{{n}_{i}}+{g}_{Ii}^{{n}_{i}}}\bullet \sum _{j\in activators}{\omega }_{j}\frac{{g}_{Aj}^{{n}_{j}}}{{K}_{j}^{{n}_{j}}+{g}_{Aj}^{{n}_{j}}},$$

where *ω*_*j*_ is the weight and *g*_*Aj*_ is the concentration of activator *j*, and *g*_*Ii*_ is the concentration of inhibitor *i*. For simplicity, all the product concentrations (*g*) were normalized to the range between 0 and 1 (Supplementary Section 1). We used equal weight for each activator (*ω*_*j*_ = 1/*n*, where *n* is the number of activation terms).

In addition, when a gene is only negatively regulated by other nodes, this rule was further subdivided into two different cases (Fig. S1B). In case A, *G* = 0 and a target gene is not expressed in the absent of activator. In case B, the target gene has a basal production rate (${F}_{k}\ne 0$). Here, we used *F*_*k*_ = 1. Thus, a gene could have expression even in the absence of activator (provided the concentration of its inhibitor is not too high). In this case, the expression of a gene is controlled by a constitutive expression promoter, or regulated by some other signaling pathways. For case B, the formula of *G* for gene with only negative regulation is modified as:

3 $${G}_{k}=\prod _{i\in inhibitors}\frac{{K}_{i}^{{n}_{i}}}{{K}_{i}^{{n}_{i}}+{g}_{Ii}^{{n}_{i}}}\bullet {F}_{k}.$$

## Results and Discussion

### Computational search for three-node networks that can decode FM and AM inputs

In order to investigate whether the transcriptional networks had the ability to reliably decode AM and FM input signals, we computationally studied all three-node transcriptional networks (Fig. 2A). TFs control target gene expression either directly or in cooperation with other transcriptional modulators. Here, we focused on identifying gene regulatory networks (motifs) that permit differential downstream gene expression in response to two distinct input dynamics, oscillatory (FM) and sustained (AM). We limited ourselves to explore networks with three nodes (Fig. 2A): one input node (TF), one output node (O), and a third node (M) that plays a regulatory role. Since we were interested in the decoding process, we eliminated networks that contained feedback regulation to TF, and in which TF was self-regulated. Thus, each network had six links in total, each of which could be in one of three states–activation, inhibition or no regulation–so there were a total of 3^6^ = 729 networks. We further eliminated trivial networks without direct and indirect (*via* M) links from TF to O, and degenerate networks lacking regulation from M to O (these were equivalent to direct regulation from TF to O). We included the two two-node networks (TF directly positively or negatively regulating O) for the purpose of comparison. In the end, 434 topologies remained in our search. For each network, we randomly sampled 10,000 sets of parameters in the computational simulations (Fig. 2B). For each parameter set, we simulated the network for long enough time to reach steady-state for both AM and FM inputs, whose strengths had equal total area under the input curves (Fig. 2B). For the AM input, the steady-state gene expression level was directly read out, while for the FM input, it was defined as the mean level of the final steady oscillatory profile (Fig. 2C). Here, we defined a parameter set as “responsive” when the average output level was >0.1 for AM or FM input when TF is “On”, and <0.1 when TF is “Off”. Furthermore, we defined a “responsive” parameter set as “AM-responsive” when the output for the AM input was at least five times larger than that of the FM input, and “FM-responsive” for the converse. Note that by “AM- or FM-responsive” it does not imply that the network can only respond to AM or FM signal. It is a measure of *differential* response to AM and FM inputs. Also note that many networks can respond to both AM and FM signals (with little differentiation ability) (Supplementary Fig. S9).

For each network, we calculated the AM- and FM-response Q values (defined as the ratio of the number of FM- and AM-responsive parameter sets to the total number of parameter sets, respectively). A larger Q value for AM-response indicated a better ability to respond to AM input, while a larger Q value for FM-response indicated a better ability to decode FM input. The Q-value can be viewed as a measure of the robustness of functional networks in biological systems under internal and environmental noise and fluctuations. Here, we define a network as “AM-responsive network” if its AM-response Q-value was larger than 20% of the maximum Q-value for AM-responsive sets one network can attain, and “FM-responsive network” for the converse. Moreover, if a network had large Q values for both AM- and FM-responsive sets, it was able to decode both types of input (with different parameters). Simulations carried out with other input conditions, different the number of parameters sets, and different criteria of “responsiveness” yielded similar results (Figs S2–S6).

### Network topologies that reliably decode FM and AM inputs

**Figure 3:**
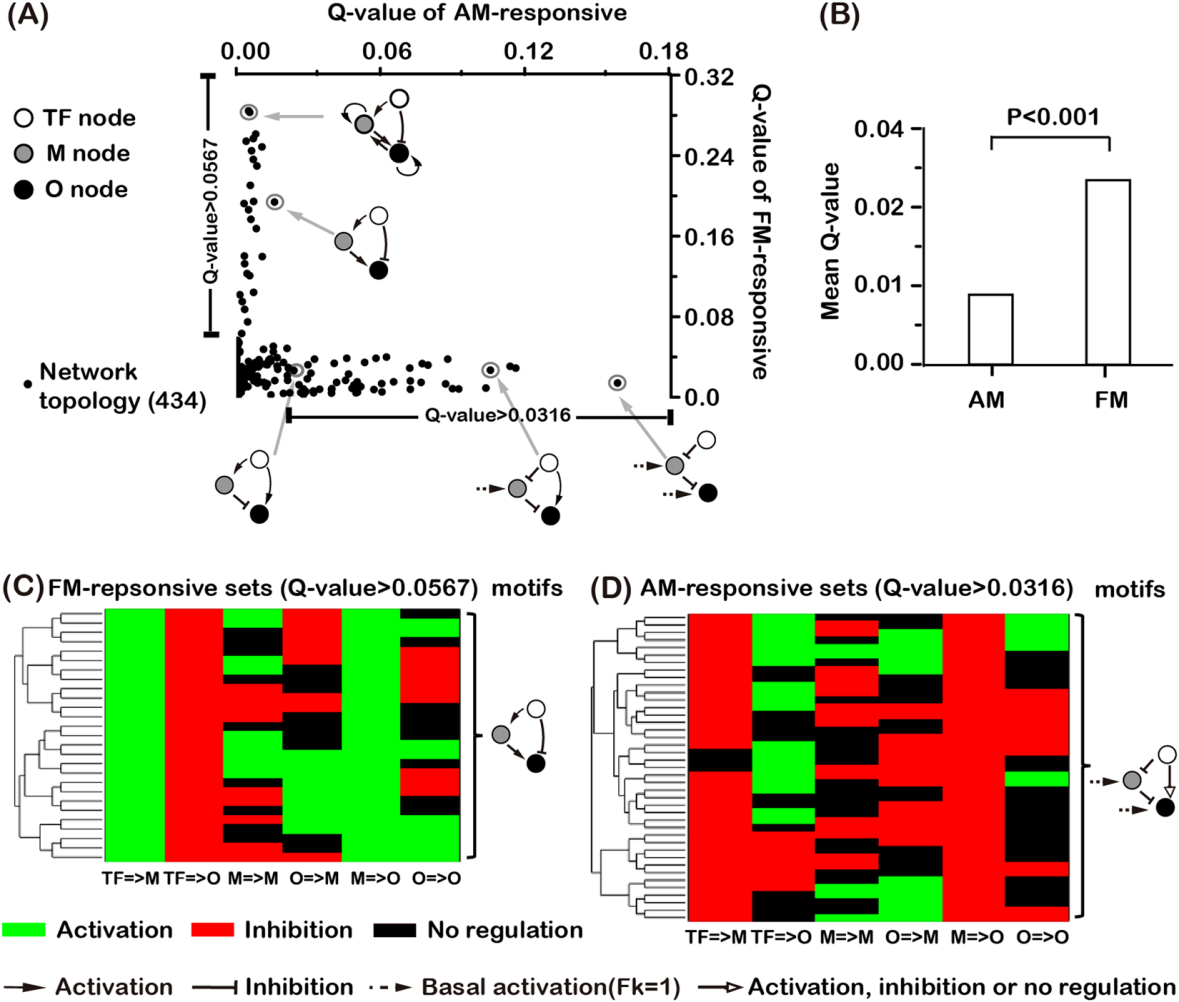
Network’s ability and typical motifs to decode AM and FM inputs. (**A**) Scatter plot for the Q values of AM- and FM-responsive sets. Each point represents one of the 434 networks. Also shown are representative motifs in decoding FM and AM inputs. (**B**) The mean Q value of AM- and FM-responsive sets for the 434 networks. Student’s *t*-test was used to obtain the significance. (**C**) Clustering of networks with FM Q value >0.0567 (20% of the maximum FM Q value). Common structural features are extracted and shown on the right of the panel. (**D**) Same as in (**C**), for networks with AM Q value >0.0316 (20% of the maximum AM Q value).

Our major question was whether the transcriptional networks modeled with Eqs (1)–(3) had the ability to reliably decode AM and FM inputs. Shown in Fig. 3A are the Q values for all 434 networks (plotted with X-axis AM Q value and Y-axis FM Q value). We found that networks vary greatly in their ability to decode FM and AM inputs. Most data points were close to either X- or Y- axis, implying that most of networks can only differentially respond to either AM or FM input, but not both. Note that the Q values of AM-responsive sets were smaller than FM-responsive sets, indicating that in general networks can differentially respond to FM input over AM input much easier than the other way around (Fig. 3B). Even with a less stringent fold-difference criterion, the relative Q values for AM- and FM-responsiveness remained qualitatively the same (Fig. S4).

To further explore the structures of the networks with high Q values, we clustered the FM-responsive networks (Fig. 3C) and the AM-responsive networks (Fig. 3D), respectively, using a pair-wise distances between networks. Each column of the heat map represents one kind of specific regulation, and different rows represent different network topology. We found that almost all FM-responsive topologies contain the direct negative regulation from TF to O and indirect positive regulation from TF to O (*via* M) (Fig. 3C). Some network topologies with better performance (high Q values for FM sets) have additional positive links, which helped to accumulate the expression level of M and/or O for FM input (Fig. 3A). On the other hand, the most common feature for AM-responsive network topologies was that they all had the negative regulation from M to O and a positive regulation of O (either from TF or basal) (Fig. 3D). The common motifs shown on the right of each panel were “average” motifs. In the FM cases (Fig. 3C) it is both necessary and sufficient for the function, and in the AM case (Fig. 3D) it is sufficient but not necessary.

### The capability to distinguish between AM and FM inputs for two-node networks

**Figure 4:**
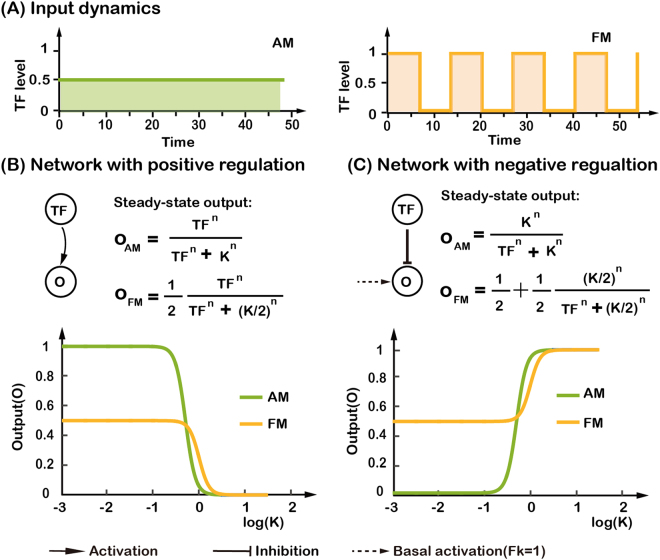
Analysis of two-node networks for decoding AM and FM input signals. (**A**) Input signals with two different transcription factor dynamics, whose strengths has equal total area under the input curves. (**B**) Steady-state expression level of the target gene for two-node network with positive regulation. It is plotted as a function of log(*K*) with *n* = 4 for FM input (yellow) and AM input (green), respectively. (**C**) Same as in (**B**), but with negative regulation.

In order to understand the mechanism of three-node networks in decoding AM and FM inputs, we carried out a theoretical analysis of two-node networks with direct regulation from TF to O (Fig. 4). The expression level of a target gene can be described by the following equation:

4 $$\frac{dx}{dt}=\frac{1}{\tau }(G({g}_{I},{g}_{A})-x),$$

where *x* represents the normalized output (O) protein concentration, *τ* is the half-life of the protein, and *G* represents the regulatory effect: either $G=\frac{T{F}^{n}}{T{F}^{n}+{K}^{n}}$ or $G=\frac{{K}^{n}}{T{F}^{n}+{K}^{n}}$ for positive or negative regulation to the output node.

First, we assessed the effect of direct positive regulation. With the AM input, the steady-state of output gene expression can be easily obtained:

5 $$x=\frac{T{F}^{n}}{T{F}^{n}+{K}^{n}}$$

With the FM input, the average of crest and trough of the steady-state output gene expression level can also be obtained (see Supplemental Materials for details):

6 $$x=\frac{1}{2}\frac{T{F}^{n}}{T{F}^{n}+{(K/2)}^{n}}.$$

Strikingly, we found that the average output level with FM input was independent of the frequency of the input oscillation and was only determined by the activation threshold *K* and the Hill coefficient *n*. (Note that this frequency independence is due to the particular form of the FM input signal used. If we use a form in which each pulse of TF has a fixed width, the output level can be proportional to the input frequency (Supplementary Fig. S10))^6,13^. The output with an AM input was larger than that with an FM input in most parameter ranges (Fig. 4B). However, it would be difficult to reliably distinguish between AM and FM inputs due to the small difference in outputs (only two-fold; smaller than our criterion five-fold). Note that there exists a small parameter range (between 0 and 1), in which the output with FM input is larger than that with AM input (Fig. 4B). Within an even narrower parameter range (0.05 < log(*K*) < 0.15), the two-node network with positive regulation can reliably decode FM input (with an output difference larger than five folds than AM and an output level larger than 0.1).

For direct negative regulation from TF to O, the steady-state output with the AM input is

7 $$x=\frac{{K}^{n}}{T{F}^{n}+{K}^{n}}.$$

With the FM input, the steady-state output is

8 $$x=\frac{1}{2}+\frac{1}{2}\frac{{(K/2)}^{n}}{T{F}^{n}+{(K/2)}^{n}}.$$

Contrary to the case of direct positive regulation, here, the AM output was smaller than the FM output for most parameters. More interestingly, the network with negative regulation had a large FM/AM ratio of outputs for a wide range of parameters, indicating its good ability to decode the FM input (Fig. 4C). However, the negatively regulated two-node network alone was unable to decode AM and FM inputs by itself. This is because it has the largest output value (*x* = 1) with TF = 0, while we required that a valid TF responsive network should have an output smaller when TF is “Off” than when TF is “On”.

To summarize, theoretical analysis showed that the positively regulated two-node network is a poor decoder for AM inputs, being able to generate at most two-fold difference between AM and FM signals (Fig. 4B), and that the negatively regulated two-node network cannot by itself be used to differentially respond to either AM or FM input. We obtained the similar results with an unnormalized version of Eq. (4) (see Supplemental Materials for details). The situation was greatly improved by adding one extra node as we have seen from the previous section – there were 41 AM responsive networks with the Q value larger than 20 percentage of the maximum AM Q value (Fig. 3A) and the indirect positive regulation from TF to O (*via* M) helped the negatively regulated two-node network become an excellent FM decoder.

### Mechanism of decoding AM and FM inputs

In order to investigate how some of the three-node networks gained the ability to decode AM and FM signals, we analyzed their functional parameters. There were eight parameters for the common motifs (Fig. 3C and D): the protein degradation rate 1/*τ* for M and O, the half-activation or half-repression threshold *K* and the Hill coefficient *n* for each one of the three edges. The parameters 1/*τ* and *K* were uniformly sampled in the logarithmic scale and the parameter *n* was uniformly sampled in linear scale, using Latin Hypercube sampling method, in our computational simulations^33^, so a non-uniform distribution of the responsive parameter sets would indicate that this parameter had constraints for decoding input signals (see Supplemental Materials for details).

**Figure 5:**
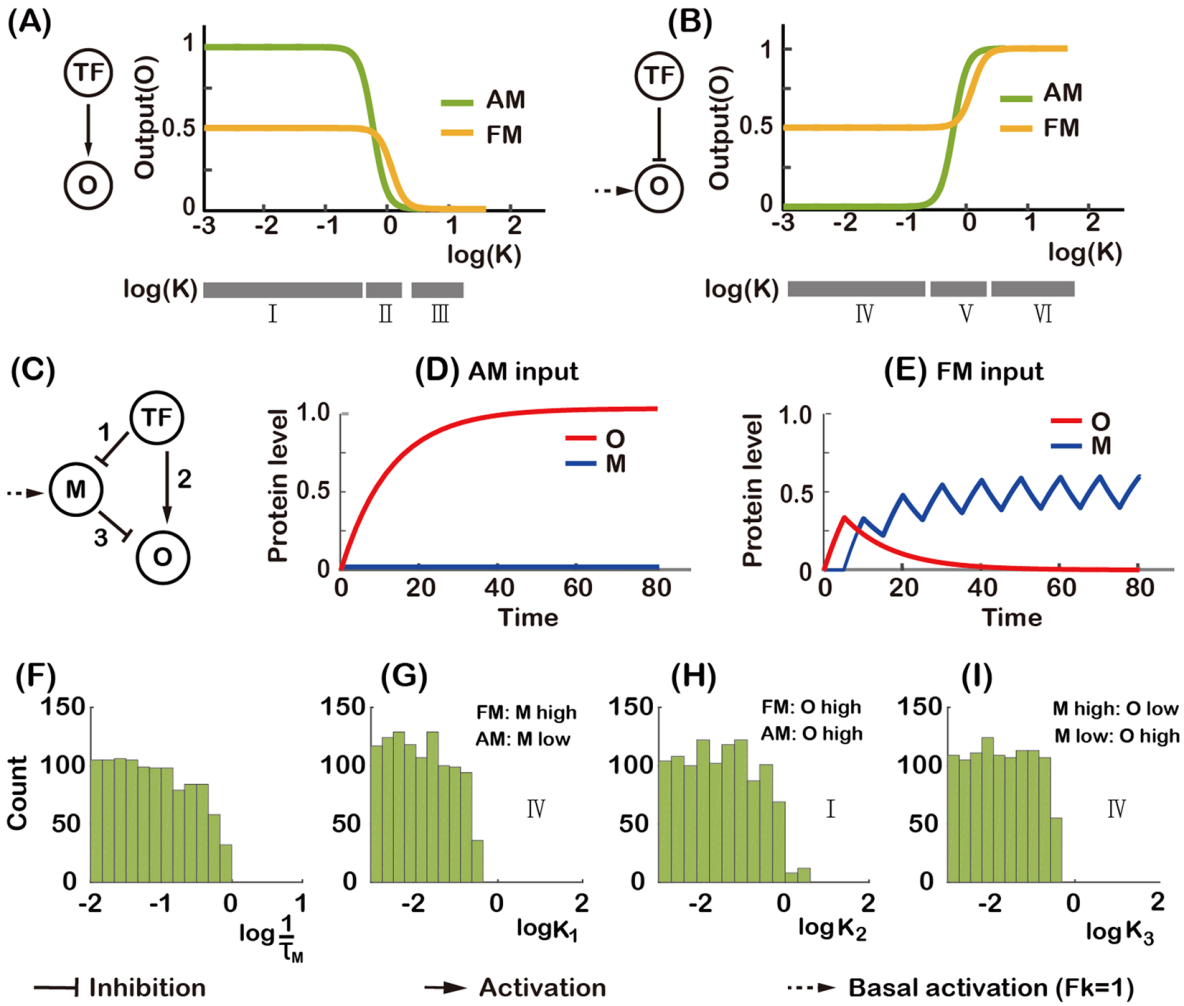
Analysis of AM-responsive topology. (**A,B**) Steady-state expression level of target gene for two-node networks (the same as in Fig. 4). The range of parameter *K* is subdivided into different regions as labeled. (**C**) An AM-responsive network with numbered links. (**F**–**I**) The histograms of functional parameters. (**F**) Parameter *τ*_*M*_ is the half-life of the gene product of node M. (**G**–**I**) Parameter *K*_*i*_ is the activation or repression threshold of the *i*^th^ link. Roman numerals indicate the parameter regions of two-node networks in Fig. 5A and B. The distributions of the other functional parameters do not differ significantly from the uniform distribution and are not shown. The combined effect of the parameters is that the product of node M can accumulate for FM but not for AM input, and can thus repress the expression of O for FM but not for AM input (**D** and **E**). (**D** and **E** were generated with $1/{\tau }_{M}=1/{\tau }_{o}=0.08$, *K*_1_ = *K*_2_ = *K*_3_ = 0.01 and *n*_1_ = *n*_2_ = *n*_3_ = 4).

For an AM-responsive network shown in Fig. 5C,we found that the parameter 1/*τ* for M and the parameter *K* for each one of three edges had constraints (Fig. 5F–I). Other parameters, which did not differ from the uniform distribution, were not shown (Fig. S7). Because the functional parameters did not have any significant pairwise correlations (Fig. S8) and the constraints on these parameters were similar to that of two-node networks (Fig. 5A,B), we used the analysis of two-node networks to understand the mechanism of three-node networks decoding input signals. Here, the functional parameter ranges for *K* is subdivided into different regions as labeled (Roman numerals: to be compared with the lower bar in Fig. 5A and B). For the network topology shown in Fig. 5C, a small value of *K*_2_ (Fig. 5H) led TF to induce the expression of O for both AM and FM inputs (region I in Fig. 5A). And TF repressed the expression of M with a small value of *K*_1_ for AM input (Fig. 5B and G), which switched off the indirect regulation from TF to O. Thus, TF was only an activator for O by direct regulation for AM input (Fig. 5D). While with a FM input, small value of 1/*τ*_*M*_ (Fig. 5F) and *K*_1_ (Fig. 5B and G) resulted in a high expression level of M, which repressed the expression of O (Fig. 5E). So, with the help of M, TF also acted as an inhibitor of O for FM input by indirect regulation (*via* M). Therefore, the combinatory effect of these parameters led the networks having higher expression level of O with an AM input than with an FM input.

**Figure 6:**
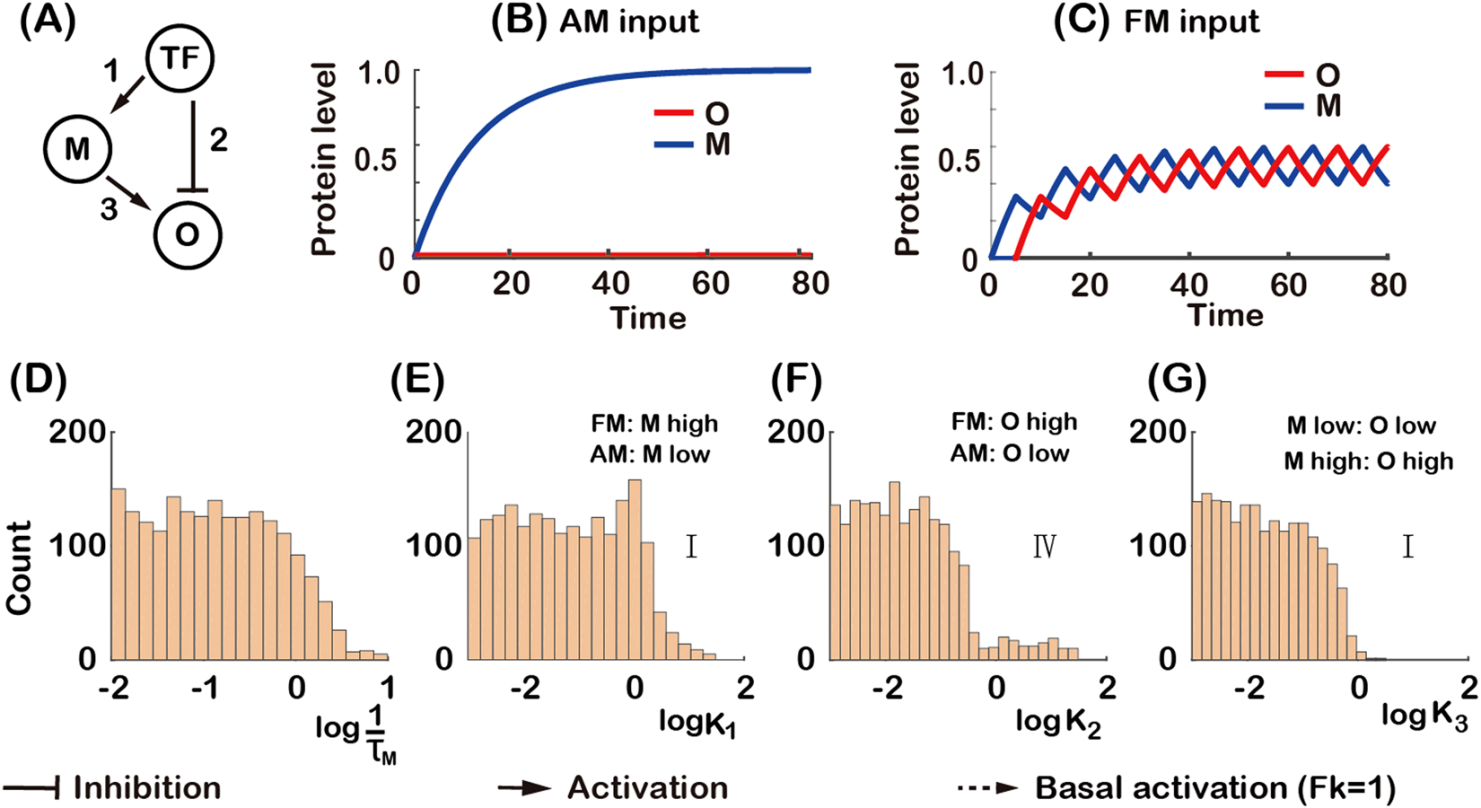
Analysis of FM-responsive topology. (**A**) A FM-responsive network with numbered links. (**D**–**G**) The histograms of functional parameters. (**D**) Parameter *τ*_*M*_ is the half-life of the gene product of node M. (**E**–**G**) Parameter *K*_*i*_ is the activation or repression threshold of the *i*^th^ link. Roman numerals indicate the parameter regions of two-node networks in Fig. 5A and B. The distributions of the other functional parameters do not differ significantly from the uniform distribution and are not shown. The combined effect of the parameters is that TF activates O indirectly through M for both AM and FM input, and TF directly represses the expression of O for AM but not for FM input (**B** and **C**). (**B** and **C** were generated with $1/{\tau }_{M}=1/{\tau }_{o}=0.08$, *K*_1_ = *K*_2_ = *K*_3_ = 0.01 and *n*_1_ = *n*_2_ = *n*_3_ = 4).

For a FM-responsive network shown in Fig. 6A, it was also only the parameters 1/*τ* for M and *K* for each one of the three edges that had constraints (Fig. 6D–G). A small value of *K*_1_ (from TF to M) and *K*_3_ (from M to O) (Fig. 6E and G) led TF to induce the expression of O by indirect regulation (*via* M) for both AM and FM inputs (region I Fig. 5A). A small degradation rate 1/*τ*_*M*_ (Fig. 6D) enabled the expression of M to accumulate and maintain at a high level. At the same time, a small value of parameter *K*_2_ (from TF to O) (Fig. 6F) led TF to repress the expression of O for AM input but not for FM input (region IV in Fig. 5B), which resulted in a low expression level of O for AM input (Fig. 6B) and a high expression level of O for FM input (Fig. 6C). Thus, TF acted as an activator for O by indirect positive regulation (*via* M) for both AM and FM inputs, but the direct negative regulation of O by TF eliminated AM response and kept FM response (region IV in Fig. 5B).

Taken together, most functional networks had two routes from TF to O (direct and indirect (*via* M)). They shared the same mechanism to differentially decode AM or FM signal. Specifically, TF activated O in one route but inhibited O for the unwanted type of input (AM or FM) in another route. For AM decoder, TF acted as an inhibitor of O by promoting the expression of M for FM input. For FM decoder, TF directly inhibited O for AM input. One exception is the motif shown in the lower right corner of Fig. 3A, in which there is only one pathway from TF to O. In this case, the double negative served as activation for AM, but not for FM due to the M node which could still have a substantial expression with FM signal.

**Figure 7:**
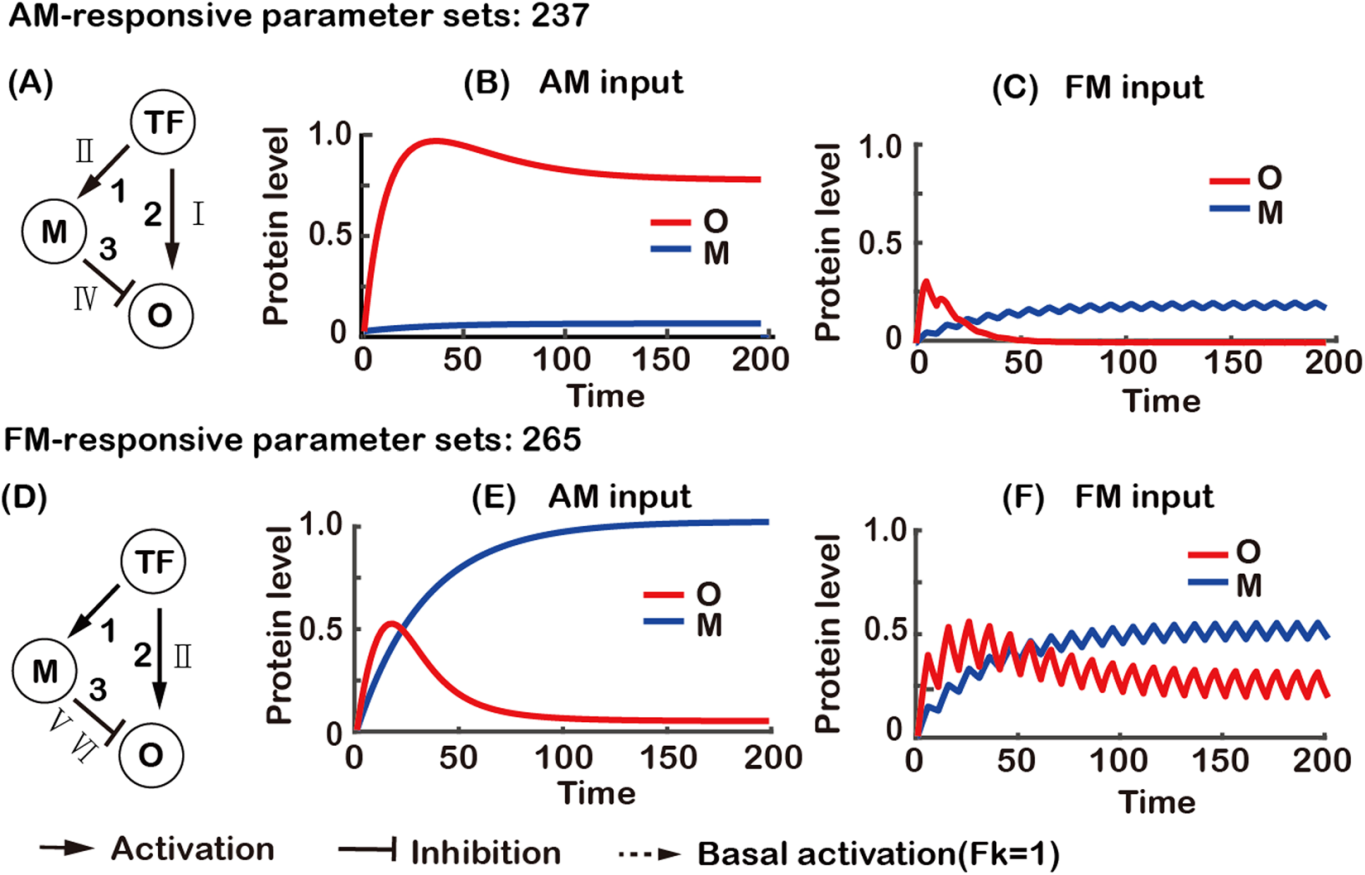
Analysis of a topology that can respond to both AM and FM inputs. (**A**) Parameter analysis for responding to AM input. With the parameters *K*_*i*_ of the links in the appropriate regions as indicated in Fig. 5A and B. TF induces a higher expression of M for FM input than AM input, resulting in higher expression of O for AM input (**B**) and low expression of O for FM input (**C**). (**B** and **C** were generated with $1/{\tau }_{M}=0.03$, $1/{\tau }_{o}=0.1$, *K*_1_ = 1.12, *K*_2_ = *K*_3_ = 0.05 and *n*_1_ = *n*_2_ = *n*_3_ = 4). (**D**) Parameter analysis for responding to FM input. With the parameters *K*_*i*_ of the links in the appropriate regions as indicated in Fig. 5A and B. When parameter *K*_3_ (from M to O) was in the region V, parameter *K*_1_ (from TF to M) had to be in the region I. TF induces a higher expression of M with AM (**E**) than FM input (**F**), resulting in a higher expression of O for FM input (**F**) and a lower expression of O for AM input (**E**). When parameter *K*_3_ was in the region VI, which makes the inhibition to O by M essentially nonexistent, this network is reduced to a two-node network. (**E** and **F** were generated with $1/{\tau }_{M}=0.03$, $1/{\tau }_{o}=0.1$, *K*_1_ = 0.01, *K*_2_ = 0.4, *K*_3_ = 0.5 and *n*_1_ = *n*_2_ = *n*_3_ = 4).

From the Q-value scatter plot shown in Fig. 3A, some networks had an ability (though weak) to decoding both AM and FM inputs, presumably by using different values of parameters. One example is shown in Fig. 7A and D for AM and FM decoding, respectively. When functioning as an AM-responsive network (Fig. 7A), the parameter *K* for the direct positive regulation from TF to O was in the region I, so TF activated O for both AM and FM inputs (Fig. 5A). The differentiation between AM and FM inputs was accomplished by the node M, the positive regulation of which by TF was in the parameter region II (Fig. 5A), so that M was more sensitive to FM than AM input. With the negative link from M to O, this difference in the values of M made O to respond to AM but not FM input (Fig. 7B and C). When functioning as an FM-responsive network (Fig. 7D), the parameter for the negative regulation from M to O could be in two different regions. When it was in the region VI, a large value of *K*_3_ removed the effect of indirect regulation from TF to O, and the network degenerated to the two-node network with direct positive regulation from TF to O (the same can be achieved by letting *K*_1_ work in region III), which resulted in O having higher expression level with FM input than with AM input for a value of *K*_2_ in region II (Fig. 5A). When the parameter *K*_3_ was in the region V, the parameter for the positive regulation from TF to M had to be in the region I, which resulted in a higher expression of M with AM than FM input (Fig. 5A). With the combined effect of negative link from M to O and a value of *K*_2_ in the region II, the network had a larger response to FM than AM input (Figs 5A, 7E and F).

### Summary

Expanding numbers of studies have documented that cells encode upstream information in the form of the temporal dynamics of TFs. And downstream genes can decode TF dynamics accordingly to elicit suitable responses^1,7,9^. While the encoding process is relatively clear, the decoding process remains largely unknown. In this study, we investigated if and how the transcriptional network topology could facilitate this decoding ability. We found that two-node networks had very limited ability in differentiating AM and FM inputs. With the help of an extra regulatory node M, three-node transcriptional networks could have excellent performance in decoding AM and FM input signals. This was in general achieved by coordinating two routes of signal transmission from input to output, in which one route activates the output while the other route plays the role of differentially repressing the unwanted type of signal input (AM or FM). These results were rather robust and independent of the choice of specific input conditions (Figs S2–S6).

Although two-node networks by themselves were not very good differential decoders, their analysis served as a base to understand the mechanisms of decoding in larger networks. The analysis revealed that the half-activation or half-repression threshold *K* played a crucial role in enabling the three-node networks to decode AM and FM inputs. By coordinating the working range of *K* in each link, the right motifs can function as robust AM or FM decoders. The parameter *K* is the ratio of unbinding to binding rates of the TF with the promoter. These rates are easily tunable either by evolution or promoter design, suggesting that the network decoding mechanisms found here could be readily implemented in nature and in synthetic biology.

## Electronic supplementary material

Supplementary Information
