# Supplementary material for: Network Motifs Capable of Decoding Transcription Factor Dynamics
Source: Sci Rep. 2018 Feb 26;8:3594. doi: 10.1038/s41598-018-21945-2 (PMC5827039; doi:10.1038/s41598-018-21945-2)
Supplement: Supplementary file 1 — Supplementary Information [file 41598_2018_21945_MOESM1_ESM.pdf]

Supplementary Materials

Network Motifs Capable of Decoding Transcription Factor Dynamics

Zongmao Gao, Siheng Chen, ShanShan Qin, Chao Tang

Simulations

Before simulations, we normalize equations for M and O based on previous methods that can reduce the parameter space[1]. For example, consider the two-node network with activation from TF to output node (*O*):

$$\frac{do}{dt} = v_o \frac{TF^n}{TF^n + K^n} - \frac{1}{\tau} o, \tag{S1}$$

If we let

$$x = \frac{1}{v_o \tau} o, \tag{S2}$$

where $v_o \tau$ is the maximum intensity of the output gene in real (unnormalized) concentration. Substituting the Eqs. (2) into Eqs. (1), we can obtain the normalization equation:

$$\frac{dx}{dt} = \frac{1}{\tau}\left(\frac{TF^n}{TF^n + K^n} - x\right). \tag{S3}$$

In the simulation, we use the Latin Hypercube sampling method to uniformly (in logarithmic or linear scale, depending on the type of parameters) sample 10,000 parameter sets for each network topology. Latin Hypercube sampling method is a general method to random sampling parameters sets[2,3]. Larger number of parameter sets are also tested, but it does not change the results significantly (Fig. S6). Based on previous studies and experimental data[1,4,5], the ranges of parameters are K ~ 0.001-$10^{1.5}$ (sampled in logarithmic scale), τ ~ 0.1-100 (in logarithmic scale), and n ~ 1-10 (in linear scale, only integer values).

For one set of parameters, the ODE simulations only stops when the criteria for steady output are satisfied. For the AM input, we first calculate the change of levels in 10 time-steps for each node ΔM and ΔO. Then we calculate $\sqrt{\Delta M^2 + \Delta O^2}$. When its values smaller than 10^{-4}, we assume that it has arrived at steady state. The level of O at the current time step is recorded as the output. For the FM input, since the output is oscillating, we check the difference between the peak value in current cycle and that of the previous cycle. If the peak-peak difference is smaller than 10^{-4} (and the

trough-trough difference is smaller than 10^{-4}), it is considered to have reached a steady state. We used the integral average of the current cycle as the output.

Previous studies show that inhibition in transcription gene regulation usually has a stronger effect than (overrides) activation[6,7]. In "strong inhibition" rule, we sum up all the activation terms and multiply with equal weights ($\omega = 1/n$ where n is the number of activation terms) to ensure proper normalization.

Analytical solution for two-node network topology with normalized equation

For a two-node network topology with positive regulation from TF to output node, we can write the normalized differential equation as:

$$\frac{dx}{dt} = \frac{1}{\tau}\left(\frac{TF^n}{TF^n + K^n} - x\right), \tag{S4}$$

where x is the output level, τ is the half-life of the protein, TF is the concentration of transcription factor, K is the half-activation threshold, and n is the Hill coefficient.

With AM input, we set the input level as 1TF and obtain the steady-state output by setting the left-hand-side to zero:

$$x = \frac{TF^n}{TF^n + K^n}. \tag{S5}$$

With FM input, input alternates between 0 and 2TF (Fig. 2B). We assume that after a long time, the FM output approaches a "stable" oscillation regardless of the initial conditions with the period of 2T. When the input level starts with 2TF, and the initial value of output $x(0)=a$, the output level after the half-period time T:

$$x(T) = \frac{TF^n}{TF^n + (K/2)^n}(1 - e^{-\frac{T}{\tau}}) + ae^{-\frac{T}{\tau}}. \tag{S6}$$

After time T, the input level becomes 0. Under this condition, the initial output is x(T) and the equation is

$$\frac{dx}{dt} = \frac{1}{\tau}(-x). \tag{S7}$$

The solution of this equation can be obtained after the remaining half-period time T:

$$x(2T) = x(T)e^{-\frac{T}{\tau}}. \tag{S8}$$

So after one period time 2T, the output is

$$x(2T) = e^{-\frac{T}{\tau}}[\frac{TF^n}{TF^n + (K/2)^n}(1 - e^{-\frac{T}{\tau}}) + ae^{-\frac{T}{\tau}}]. \quad \text{(S9)}$$

By solving x(2T) = x(0), we have

$$a = \frac{e^{-\frac{T}{\tau}}}{1 + e^{-\frac{T}{\tau}}} \frac{TF^n}{TF^n + (K/2)^n}. \quad \text{(S10)}$$

If we simply calculate the average of the crest and the trough of the output protein level, we have the average output

$$\bar{x} = \frac{x(0) + x(T)}{2} = \frac{a + \frac{TF^n}{TF^n + (K/2)^n}(1 - e^{-\frac{T}{\tau}}) + ae^{-\frac{T}{\tau}}}{2}. \quad \text{(S11)}$$

From Eqs. (S10) and (S11), we have

$$\bar{x} = \frac{1}{2} \frac{TF^n}{TF^n + (K/2)^n}. \quad \text{(S12)}$$

Similarly, we can obtain the output of the network topology with direct inhibitory regulation only. With AM input signal, the steady-state output is

$$x = \frac{K^n}{TF^n + K^n}. \quad \text{(S13)}$$

The average of steady-state output with an FM input is

$$\bar{x} = \frac{1}{2} + \frac{1}{2} \frac{(K/2)^n}{TF^n + (K/2)^n}. \quad \text{(S14)}$$

Analytical solution for two-node network topology without normalized equation

For two-node network topology with positive regulation from TF to output node, the gene expression can be described as follow ordinary differential equation:

$$\frac{dx}{dt} = v_x \frac{TF^n}{TF^n + K^n} - \frac{1}{\tau} x, \quad \text{(S15)}$$

where the parameter v_x is the maximum production rate, and τ is the half-life of the protein, TF is the concentration of transcription factor, K is the half-activation threshold, and n is the Hill coefficient.

With AM input, we set the input level as 1TF and obtain the steady-state output:

$$x = \tau v_x \frac{TF^n}{TF^n + K^n}. \tag{S16}$$

With FM input, input alternates between 0 and 2TF. The steady-state average of the crest and the trough of the output protein level is

$$\bar{x} = \frac{1}{2}\tau v_x \frac{TF^n}{TF^n + (K/2)^n} \tag{S17}$$

Similarly, for two-node network topology with direct inhibitory regulation only, the gene expression can be described as follow equation:

$$\frac{dx}{dt} = v_x \frac{K^n}{TF^n + K^n} - \frac{1}{\tau}x, \tag{S18}$$

With AM input, the steady-state output is

$$x = v_x \tau \frac{K^n}{TF^n + K^n}. \tag{S19}$$

With FM input, the average of steady-state output is

$$\bar{x} = \frac{1}{2}v_x \tau + \frac{1}{2}v_x \tau \frac{(K/2)^n}{TF^n + (K/2)^n} \quad . \tag{S20}$$

Comparing with Eqs. (S13)-(S14), Eqs. (S19)-(S20) have a common constant factor. Thus, whether the equation (1) was normalized or not, it had no effect on the output ratio of with an AM input to with a FM input ($\tau \neq 0, v_x \neq 0$) since they cancel out in the ratio.

Analytical solution the relationship between the output level and the input frequency for two-node network topology with positive regulation

For a two-node network topology with positive regulation from TF to output node, the normalized differential equation can be written as:

$$\frac{dx}{dt} = \frac{1}{\tau}\left(\frac{TF^n}{TF^n + K^n} - x\right), \tag{S21}$$

where x is the output level, τ is the half-life of the protein, TF is the concentration of transcription factor, K is the half-activation threshold, and n is the Hill coefficient.

For the oscillatory input with different number pulse (Fig. S10A), it is easy to obtain the analytical expression between the number of pulse and the output level

based on the Eqs. (S6)- Eqs.(S9):

$$x(m) = \frac{TF^n}{TF^n + K^n}(e^{\frac{D}{\tau}} - 1)\frac{e^{-(\frac{T}{\tau})} - e^{-(n+1)\frac{T}{\tau}}}{1 - e^{-(\frac{T}{\tau})}}, \tag{S22}$$

where *m* is the number of pulse in a constant duration time, D is the duration for each of pulse, T is the sum of D and gap time between two pulse.

Clustering of networks

There are 6 possible edges for a network topology. For each topology, we construct a characteristic vector composed of 6 components representing 6 edges. Each component is 1 if the corresponding edge is activation, -1 if it is inhibition, and 0 if there is no regulation. We use a hierarchical clustering method to cluster the network topologies based on the Q value of AM- or FM- responsive sets, and show the characteristic vector of each network in color (green: activation, red: inhibition, black: no regulation). The MATLAB (Mathworks Inc.) function clustergram was used to cluster these network topologies.

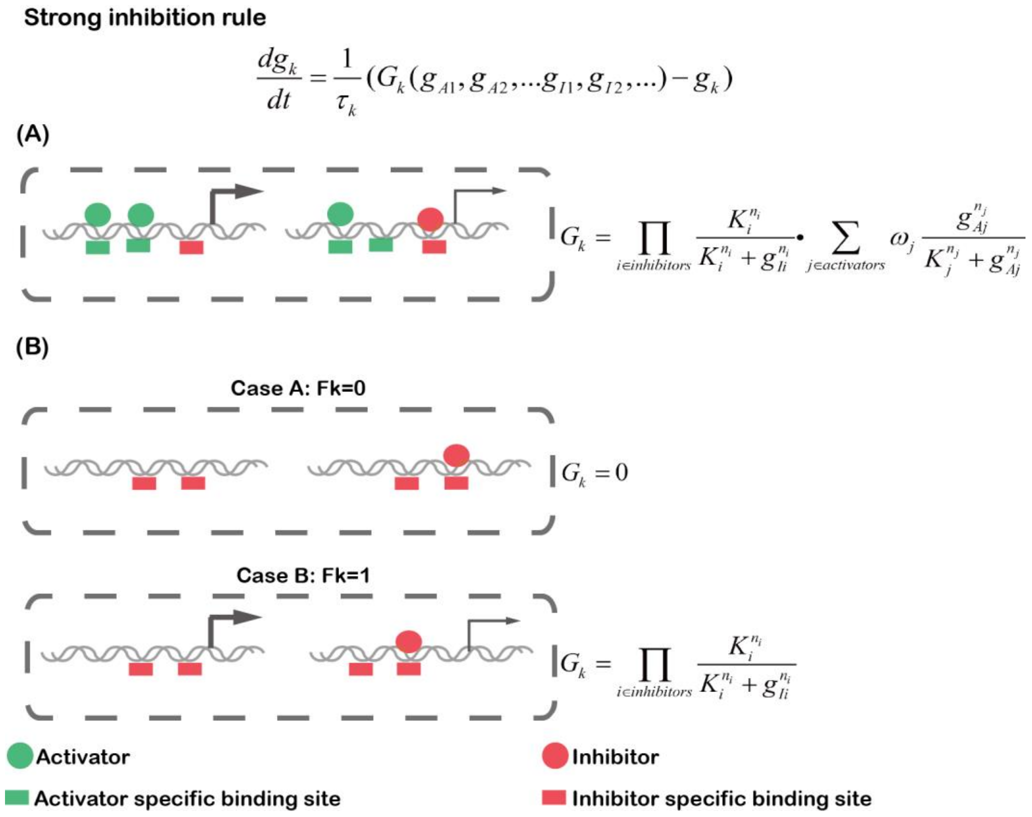

Figure S1. Modelling transcriptional regulation with strong inhibition rule

G is the overall effect of different activators and inhibitors. **(A)** A gene is regulated by both activators and inhibitors and follows the strong inhibition rule, which assumes an additive effect of activators and a strong inhibitory effect of inhibitors (multiplicative). **(B)** When the gene is only regulated by inhibitors, there are two different cases for basal level $F_k = 0$ and $F_k = 1$. F_k is the basal activation when only inhibitors exist).

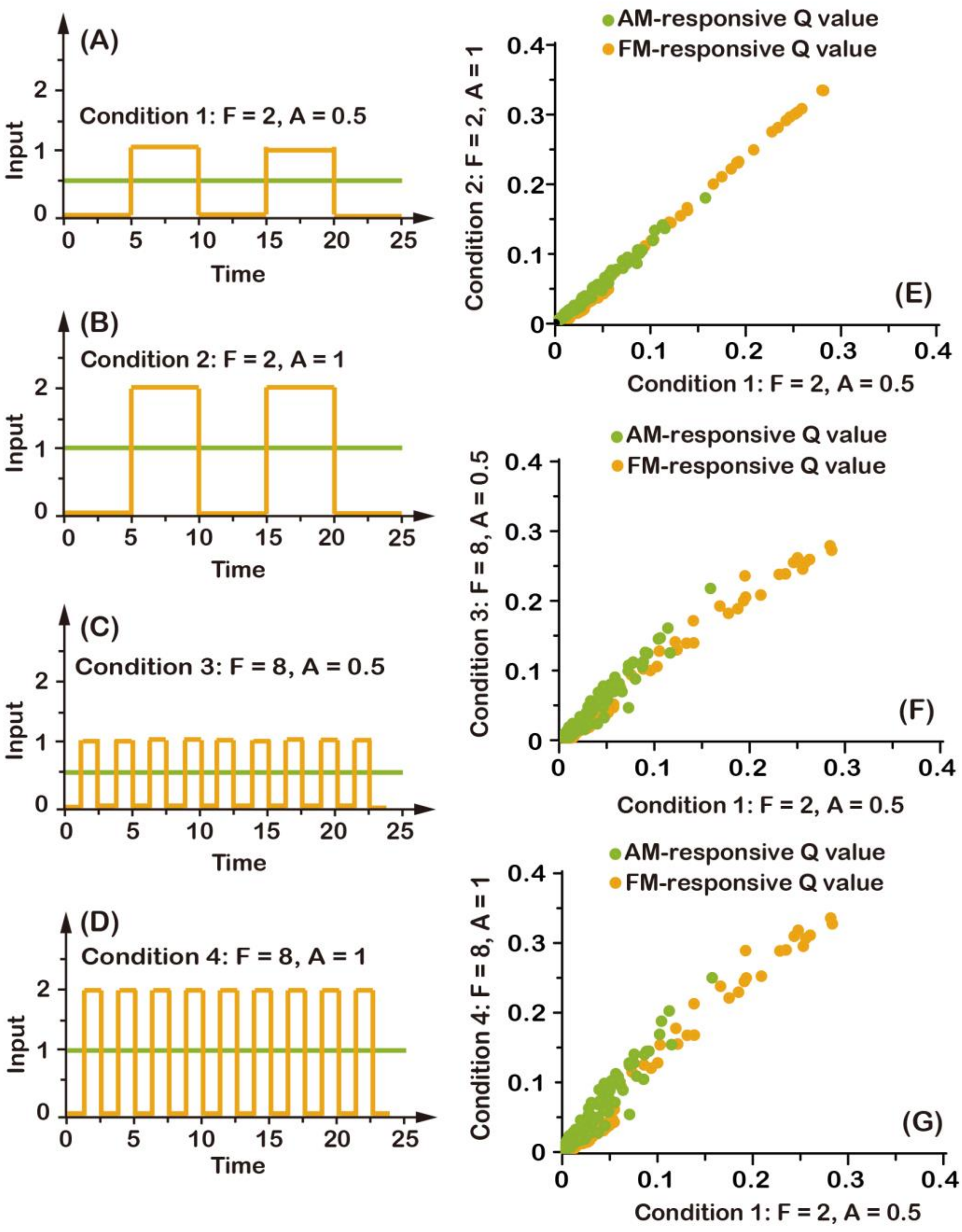

Figure S2. Simulations with different input frequencies and amplitude yield similar results.

(A)-(D) Input conditions with different frequency (F) and amplitude (A). The value of A is the intensity of TF concentration for AM input, and the value of F is the number of pulse in 20 units of time. **(E)-(G)** Scatter plots for the Q values of FM-responsive sets (yellow) and AM-responsive sets (green) for all three-node networks under two input conditions. Each point represents one kind of topology in a total of 434 networks. Most of them lie on the diagonal, indicating that these conditions do not change the results significantly.

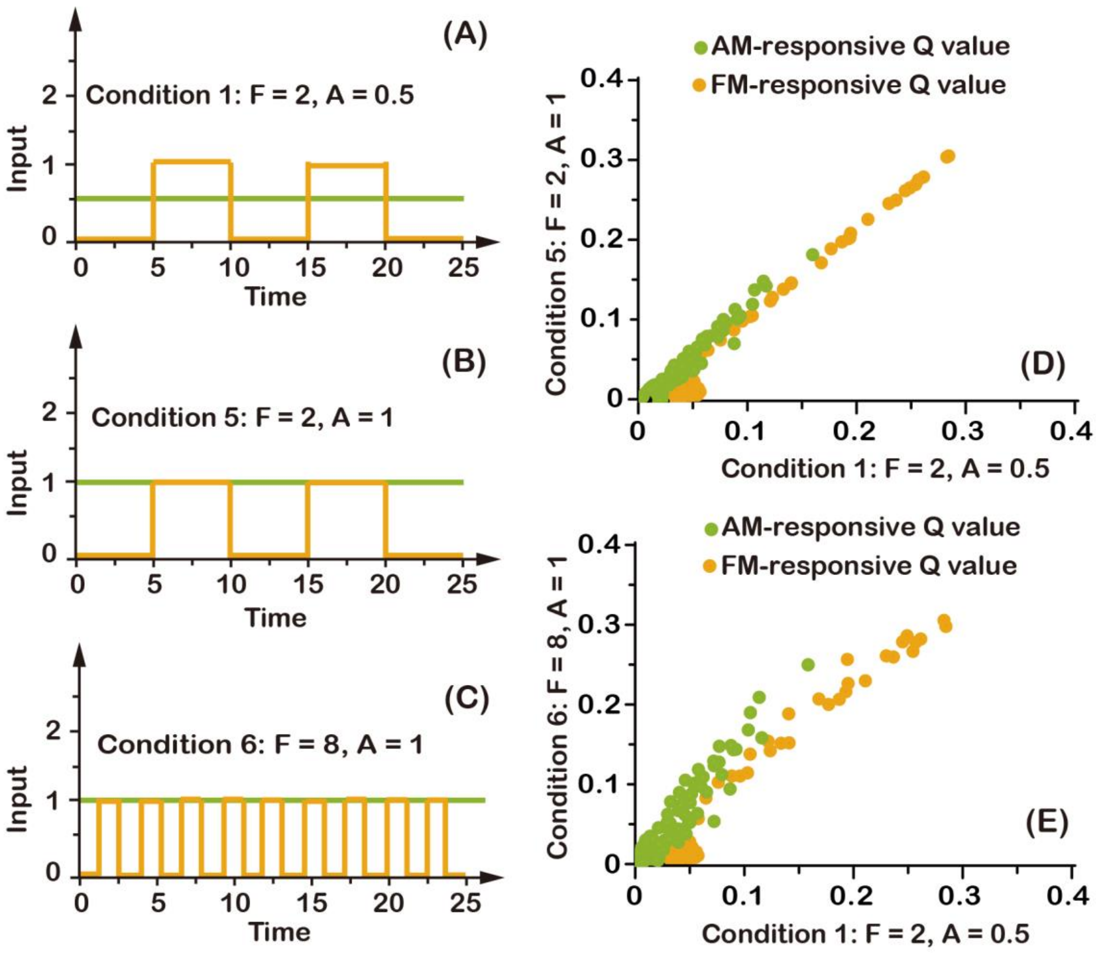

Figure S3. Simulations that were carried out with the same amplitude yielded similar results.

(A)-(C) Input conditions with different frequency (F) and amplitude (A). The value of A is the intensity of TF concentration for AM input, and the value of F is the number of pulse in 20 units of time. **(D)-(E)** Scatter plots for the Q values of FM-responsive sets (yellow) and AM-responsive sets (green) for all three-node networks under two input conditions. Each point represents one kind of topology in a total of 434 networks. Most of them lie on the diagonal, indicating that these conditions do not change the relative ability among networks significantly.

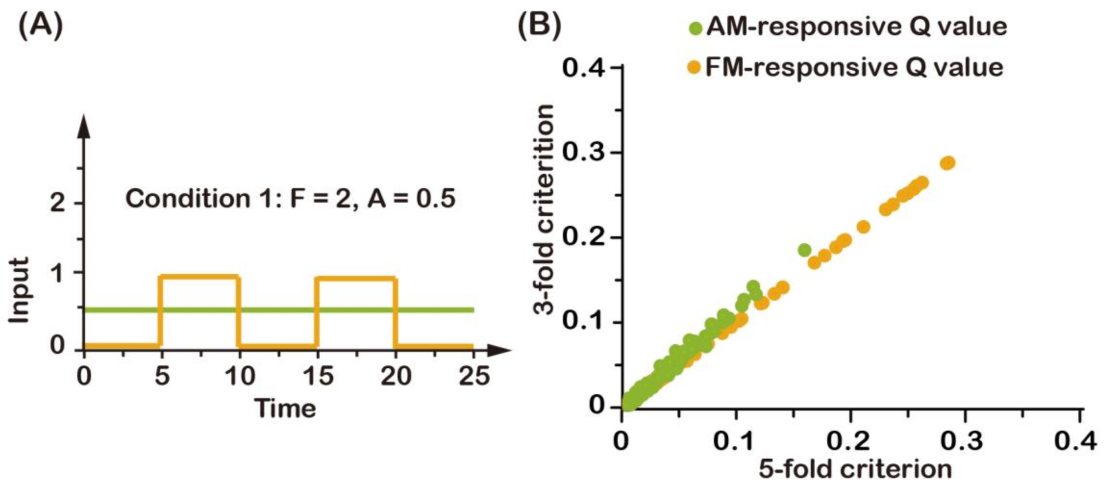

Figure S4. Simulations with two different criterions show similar results. **(A)** Simulation input condition. The value of A is the intensity of TF concentration for AM input, and the value of F is the number of pulse in 20 units of time. (**B**) Scatter plot for the Q values of FM-responsive sets (yellow) and AM-responsive sets (green) for all three-node networks with two different criterions. Each point represents one kind of topology in a total of 434 networks. Most points are close to the diagonal, which means that the criterion does not change the results significantly.

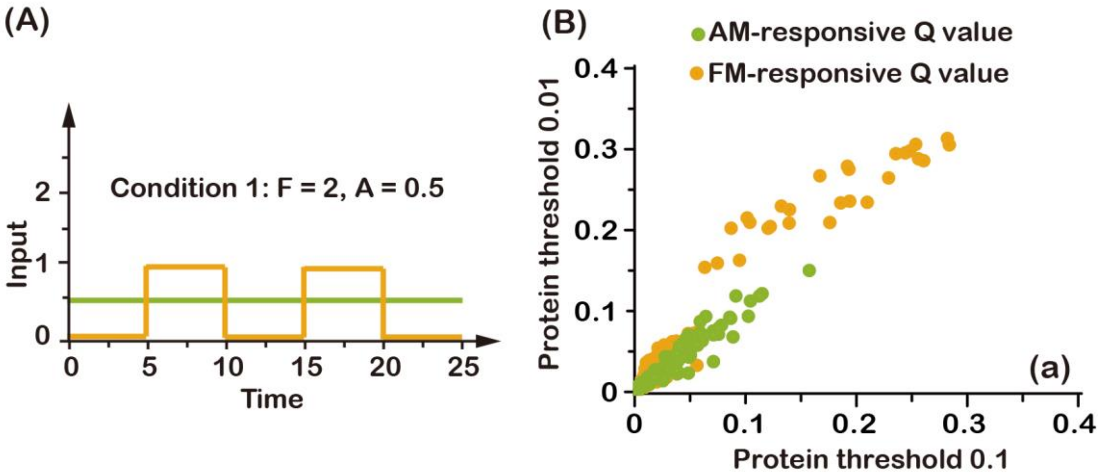

Figure S5. Simulations with two different criterions for the protein threshold show similar results.

(**A**) Simulation input condition. The value of A is the intensity of TF concentration for AM input, and the value of F is the number of pulse in 20 units of time. (**B**) Scatter plot for the Q values of FM-responsive sets (yellow) and AM-responsive sets (green) for all three-node networks with two different criterions (protein threshold level). Each point represents one kind of topology in a total of 434 networks. As can be seen, the change in the threshold does not change the results significantly.

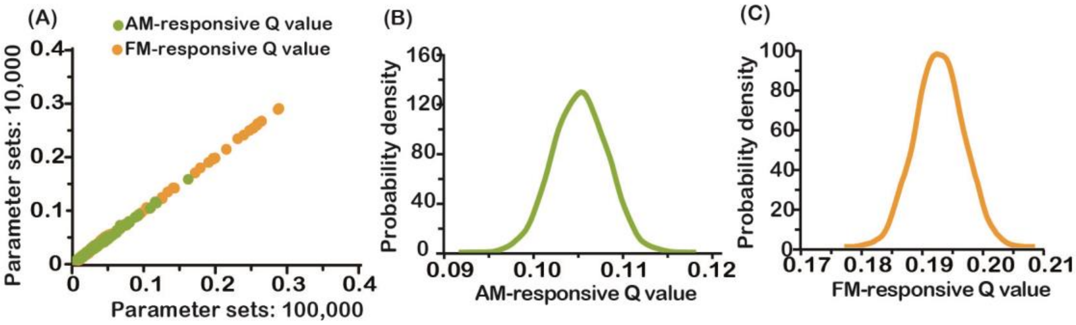

Figure S6. Simulations with different number of parameter sets.

(**A**) Scatter plot for the Q values of FM-responsive sets (yellow) and AM-responsive sets (green) for all three-node networks with two different sampling numbers of parameter sets. Each point represents one kind of topology in a total of 434 networks. (**B**) Random sampling the parameter sets 5000 times for the AM-responsive network of Fig. 5C by using the bootstrap method and the simulation results of 10,000 parameters sets. The confidence interval of 95% for the AM-responsive Q-value is [0.1052, 0.1054]. (**C**) Random sampling the parameter sets 5000 times for the FM-responsive network of Fig. 6A by using the bootstrap method and the simulation results of 10,000 parameters sets. The confidence interval of 95% for the FM-responsive Q-value is [0.1928, 0.1931].

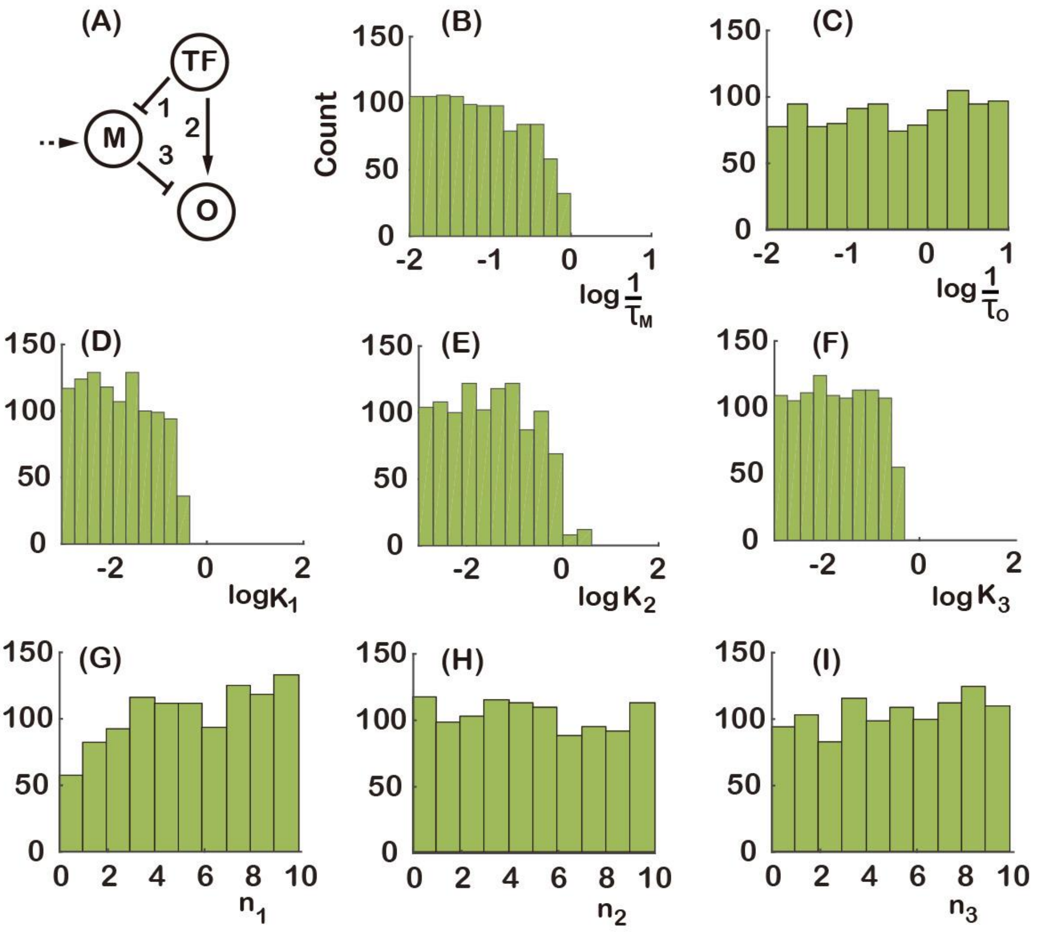

Figure S7. Parameters analysis of AM-responsive topology.

(**A**) The AM-responsive network topology of Fig. 5C. (**B**)-(**I**)The histograms of functional parameters for the network. The links are numbered. Parameter τ_M and τ_O are the half-lives of the gene products of node M and O, respectively. Parameter K_i is the activation or repression threshold of the ith link. Parameter n_i is the Hill coefficient of the ith link. As can be seen, only $1/\tau_M$ and the Ki have restrictions in their distributions. Other parameters have the same uniform distributions as initially given.

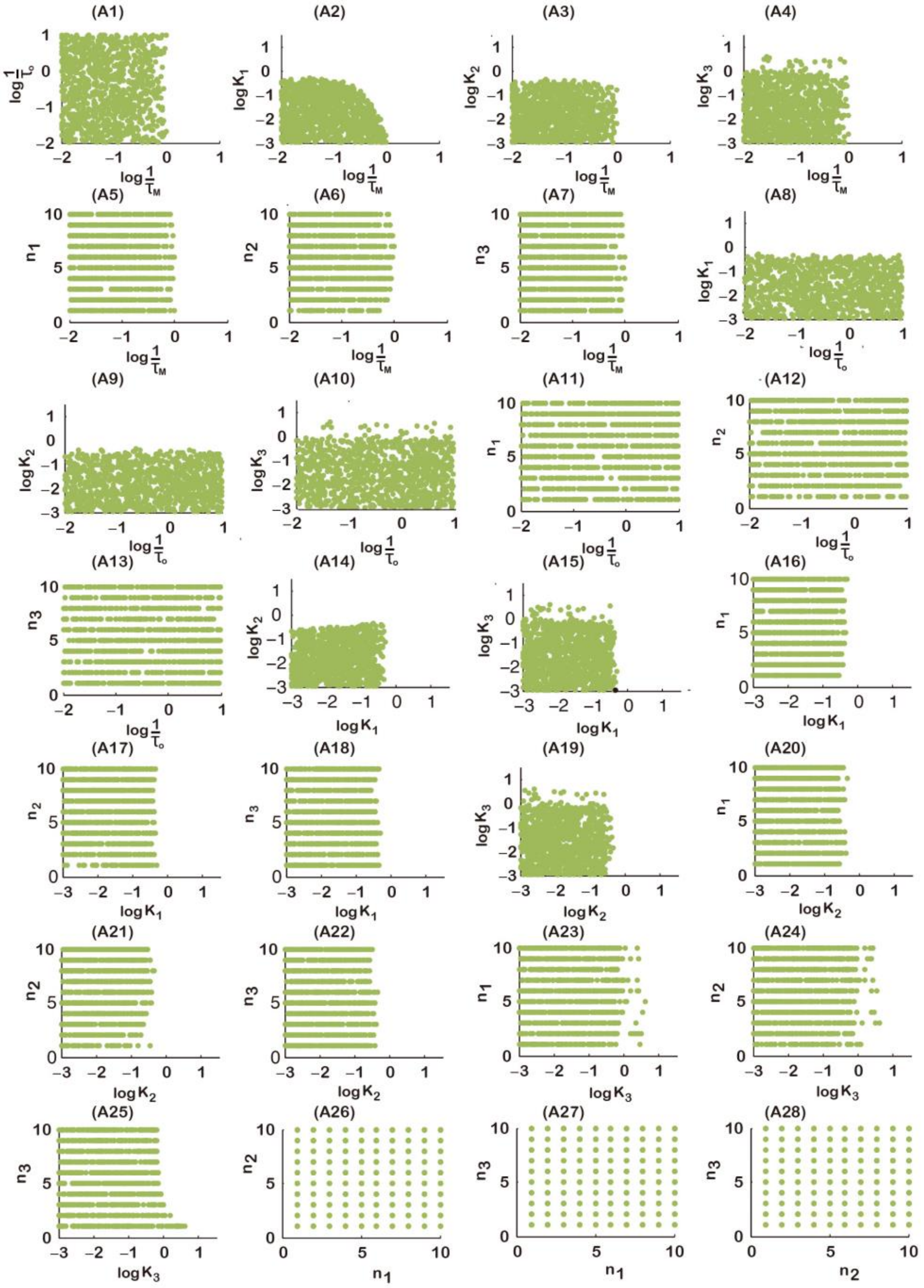

Figure S8. Scatter plot of pairwise parameter for the AM-responsive topology. (**A1**)-(**A28**) The pairwise scatter plot of the functional parameters for an AM-responsive network shown in Fig. S7. There are eight parameters: the protein degradation rate $1/\tau$ for M and O, the half-activation or half-repression threshold K and the Hill coefficient n for each one of the three edges. None of pairs showed significant correlation. Thus we can use our results in two-node networks and apply to each edge in three-node networks. The edges in three-node network thus has a sample combinatory effect, since the parameters are not correlated.

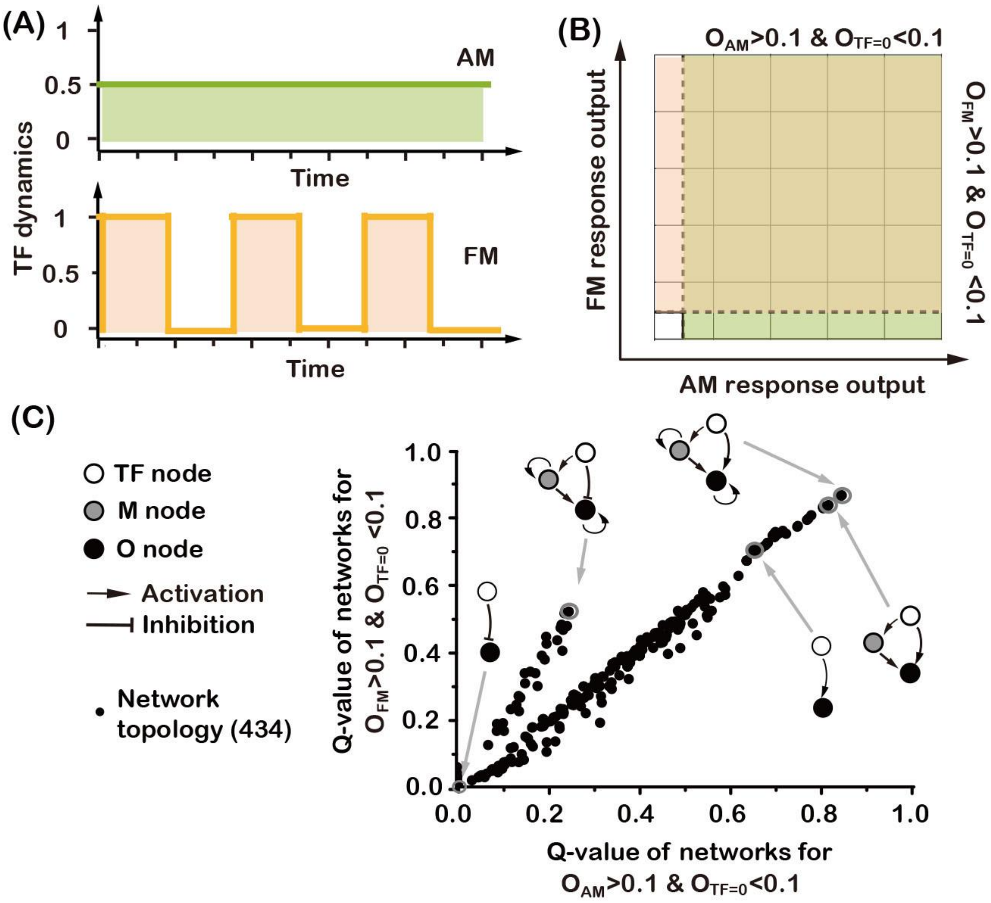

Figure S9. Searching for topologies that can response two types of transcription factor dynamics.

(A) AM and FM input dynamics of transcription factor. (**B**) Illustration of the output analysis and the criterion for response to AM- (O_{AM} > 0.1 and $O_{TF=0}$ < 0.1) or FM-input (O_{FM} > 0.1 and $O_{TF=0}$ < 0.1). For a network, a "functional parameter set" is a one with outputs falling in the dust yellow rectangle. **(C)** Scatter plot for the Q values of networks with the response criteria. Each point represents one of the 434 networks. Also shown are representative motifs.

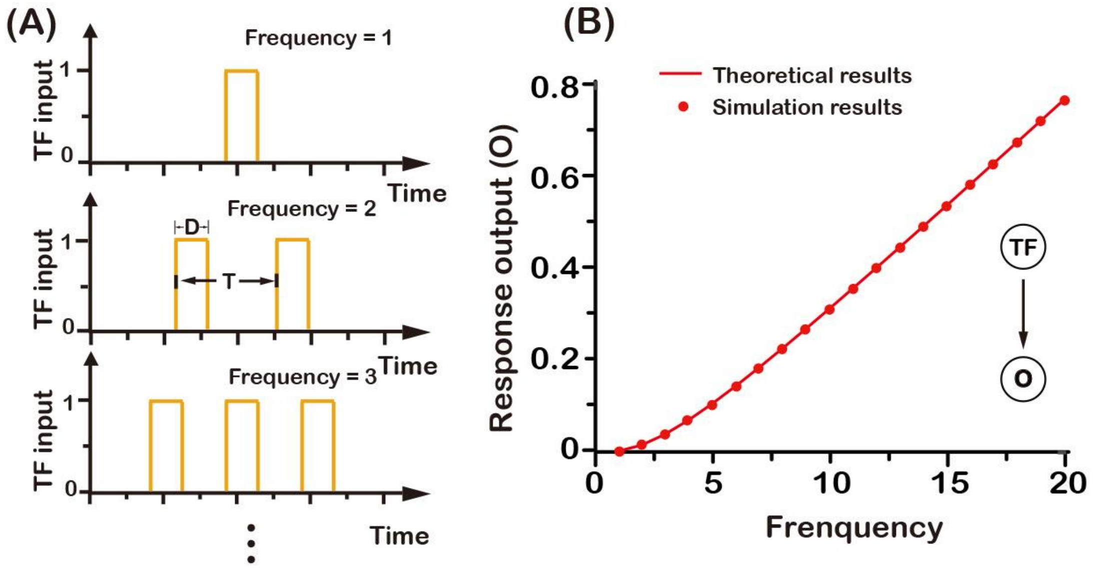

Figure S10. The relationship between frequency and the output level for two-node network with positive regulation.

(A) The input dynamics with different number of pulse within a constant time. (**B**) The relationship between the frequency (the number of pulse within the same time) and the output level for two-node network with positive regulation. Red dots represent the simulation results, while solid red line is the theoretical results. (B was generated with D = 2, total simulation time is 50, K = 0.05, n = 4, and $1/\tau_o = 0.2$.)

Reference

1. Ma, W., Lai, L., Ouyang, Q. & Tang, C. Robustness and modular design of the Drosophila segment polarity network. *Mol. Syst. Biol.* **2,** 1–9 (2006).
2. Swiler, L. P. & Wyss, G. D. A User ' s Guide to Sandia ' s Latin Hypercube Sampling Software. *SAND Rep.* 88 (1998).
3. McKay, M. D., Beckman, R. J. & Conover, W. J. A comparison of three methods for selecting values of input variables in the analysis of output from a computer code. *Technometrics* **42,** 55–61 (2000).
4. Shi, W., Ma, W., Xiong, L., Zhang, M. & Tang, C. Adaptation with transcriptional regulation. *Sci. Rep.* **7,** 42648 (2017).
5. Zong, Y. *et al.* Insulated transcriptional elements enable precise design of genetic circuits. *Nat. Commun.* **8,** 1–12 (2017).
6. Tan, N. & Ouyang, Q. Design of a network with state stability. *J. Theor. Biol.* **240,** 592–598 (2006).
7. Albert, R. & Othmer, H. G. The topology of the regulatory interactions predicts the expression pattern of the segment polarity genes in Drosophila melanogaster. *J. Theor. Biol.* **223,** 1–18 (2003).
